# Effect of MCH1, a fatty-acid amide hydrolase inhibitor, on the depressive-like behavior and gene expression of endocannabinoid and dopaminergic-signaling system in the mouse nucleus accumbens

**DOI:** 10.1590/1414-431X2024e12857

**Published:** 2024-02-19

**Authors:** C. Medina-Saldivar, G.V.E. Pardo, L.F. Pacheco-Otalora

**Affiliations:** 1 Laboratorio de Investigación en Neurociencia, Instituto Científico, Universidad Andina del Cusco, Cuzco, Perú

**Keywords:** FAAH inhibitor, Nucleus accumbens, mRNA, Endocannabinoids, Macamide

## Abstract

MCH1 is a synthetic macamide that has shown *in vitro* inhibitory activity on fatty acid amide hydrolase (FAAH), an enzyme responsible for endocannabinoid metabolism. This inhibition can modulate endocannabinoid and dopamine signaling in the nucleus accumbens (NAc), potentially having an antidepressant-like effect. The present study aimed to evaluate the effect of the *in vivo* administration of MCH1 (3, 10, and 30 mg/kg, *ip*) in 2-month-old BALB/c male mice (n=97) on forced swimming test (FST), light-dark box (LDB), and open field test (OFT) and on early gene expression changes 2 h after drug injection related to the endocannabinoid system (*Cnr1* and *Faah*) and dopaminergic signaling (*Drd1* and *Drd2*) in the NAc core. We found that the 10 mg/kg MCH1 dose reduced the immobility time compared to the vehicle group in the FST with no effect on anxiety-like behaviors measured in the LDB or OFT. However, a 10 mg/kg MCH1 dose increased locomotor activity in the OFT compared to the vehicle. Moreover, RT-qPCR results showed that the 30 mg/kg MCH1 dose increased *Faah* gene expression by 2.8-fold, and 10 mg/kg MCH1 increased the *Cnr1* gene expression by 4.3-fold compared to the vehicle. No changes were observed in the expression of the *Drd1* and *Drd2* genes in the NAc at either MCH1 dose. These results indicated that MCH1 might have an antidepressant-like effect without an anxiogenic effect and induces significant changes in endocannabinoid-related genes but not in genes of the dopaminergic signaling system in the NAc of mice.

## Introduction

MCH1 [(9Z,12Z)-N-benzyloctadeca-9,12-dienamide] is a synthetic macamide originally isolated from the *Lepidium meyenii* (1), which has demonstrated inhibitory activity of the fatty acid amid hydrolase (FAAH) in *in vitro* studies (2). The FAAH is the enzyme responsible for the biodegradation of endocannabinoids such as anandamide (AEA), which acts as an agonist of cannabinoid receptor 1 (CB1) (3). Recent studies have shown that that FAAH inhibitors can increase the AEA bioavailability, and it has been implicated in the reduction of depressive-like behavior in rodents (4-7). Increased AEA bioavailability in the nucleus accumbens (NAc) might enhance the modulatory properties of the endocannabinoid system over the altered dopamine system function associated with depression symptoms (8)

Altered dopamine (DA) system function is associated with anhedonia, which is one of the main symptoms of major depressive disorder in humans. This is consistent with animal models of depression, such as an altered dopamine release in the NAc induced by chronic mild stress (9). Moreover, acute stressors such as restraint and social defeat stress can increase DA release in the NAc (10), and these alterations are related to the establishment of more complex depressive-like behaviors (11). DA release in the NAc is subordinated to pleasurable or aversive experiences (12) and acts on its receptors D1 and D2, expressed mainly in GABAergic neurons (13,14). The DA release to the NAc is strongly regulated by glutamatergic and GABAergic projections from areas of the limbic system (9,15). In the NAc, the endocannabinoid system plays a modulatory role in maintaining a homeostatic dopamine release (16). Endocannabinoids, such as anandamide (AEA), are produced on-demand on postsynaptic GABAergic medium spiny neurons and then released into the presynaptic glutamatergic projections. AEA then interacts with the cannabinoid receptor 1 (CB1) reducing the glutamatergic transmission and, indirectly, modulating the dopamine release in NAc (15).

Due to MCH1 capacity to inhibit the FAAH activity *in vitro*, we hypothesized that MCH1 could modulate the endocannabinoid and dopaminergic signaling system in the NAc and exert an antidepressant-like effect. Therefore, we studied the effects of acute administration of MCH1 on immobility time in the forced swimming test (FST), on anxiety-related behaviors in the light-dark box (LDB), and on the open field test (OFT) in adult male mice. Moreover, we explored early changes in endocannabinoid (*Cnr1* and *Faah*) and dopamine (*Drd1* and *Drd2)* signaling-related genes in the NAc.

## Material and Methods

### Animals

Two-month-old male BALB/c mice were used for the behavioral (n=96) and molecular (n=19) experiments. Mice were housed 4-5 per cage in the animal facility of the Universidad Andina del Cusco at 24±2°C, with a diurnal cycle from 06:00 to 18:00 h, <85 dB noise, and free access to water and standard laboratory chow. All experiments were performed under the National Institutes of Health Guidelines for the Care and Use of Laboratory Animals and with the approval of the Universidad Andina del Cusco Institutional Animal Care and Use Committee (Protocol #2019-001-IACUC-1).

### Drug administration

MCH1 was synthesized by Medalchemy, S.L. (Spain). MCH1 was suspended in DMSO (Sigma-Aldrich, USA) and mixed with an equal volume of polyethylene glycol (PEG-600) (Sigma-Aldrich). Sterile 0.9% saline was added and the resulting suspension was stirred for 30 min at 50°C. The final composition of the vehicle solution was DMSO, PEG-600, and saline (1/1/18). Midazolam (MDZ, RichVet, BsAs, Argentina) was diluted in sterile 0.9% saline. Immediately after being dissolved, all drugs were administered through the intraperitoneal (*ip*) route in a volume of 10 mL/kg body weight. Fluoxetine (FLX, Sigma-Aldrich) (17) and MDZ (18) doses were selected from those reported in the literature. MCH1 doses were selected based on the ability of URB597, a well-characterized FAAH inhibitor, to increase the pharmacological effect of anandamide (19).

### Experimental design

#### Effects of MCH1 on FST, LDB, and OFT

**Figure 1:**
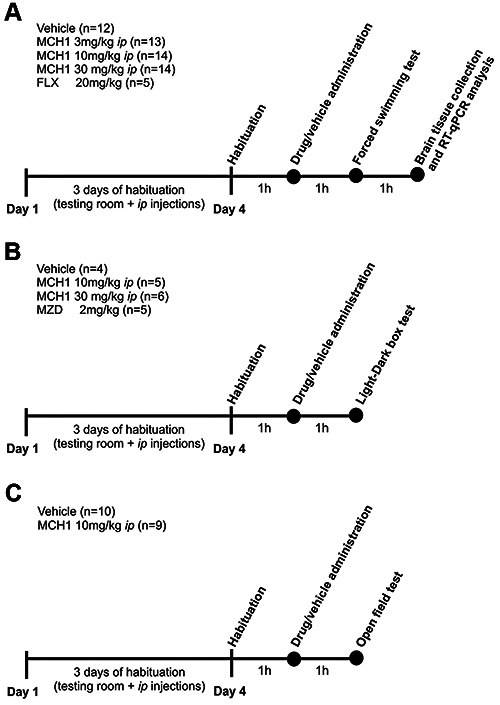
Diagram of the experimental procedure involving behavioral and molecular experiments. **A**, The first group of male BALB/c mice (n=58) received a 3-day course of vehicle solution administration for habituation to the *ip* injection and the test room. The mice stayed in the test room for one hour on the fourth day. After that, MCH1, fluoxetine (FLX), or vehicle was administered. One hour later, depressive-like behavior was evaluated using the forced swimming test, and two hours later, the whole brain was collected and stored at -80°C for molecular analysis. **B**, The second group of male BALB/c mice (n=20) underwent the same 3-day habituation procedure. On the fourth day, MCH1, midazolam (MDZ), or vehicle was administered, and one hour later, anxious-like behavior was assessed using a light-dark box test. **C**, The third group of male BALB/c mice (n=19) underwent the same 3-day habituation. On the fourth day, vehicle or MCH1 10 mg/kg was administered *ip* and, one hour later, anxiety-like behavior and locomotor activity were evaluated using the open field test. MCH1 (9Z,12Z)-N-benzyloctadeca-9,12-dienamide.

The acute effect of MCH1 on the FST, LDB, and OFT was studied in mice that were first habituated to the testing room, handling, and injection of vehicle for 3 days. One day after the last vehicle injection, animals were allowed to habituate for 1 h and then received an *ip* injection of MCH1 (3, 10, or 30 mg/kg), FLX (20 mg/kg), or vehicle (VEH). FLX was used to validate the behavioral test, but not to compare its effect with that of MCH1, as MCH1 and FLX have different pharmacokinetics and their effects cannot be directly compared. One hour later, animals were evaluated in the FST. Mice were euthanized and brains were collected one hour later. In a separate group of mice, MCH1 (10 or 30 mg/kg), MDZ (2 mg/kg), or vehicle was administered 1 h before the LDB test. In another group of mice, MCH1 (10 mg/kg) or vehicle was administered 1 h before the OFT as described above. All details of the experimental design are presented in Figure 1.

#### Effects of MCH1 on gene expression in the nucleus accumbens

Molecular analysis was performed to evaluate the effect of MCH1 on the expression of genes related to endocannabinoid and dopaminergic systems in the NAc. One hour after the FST assessment and two hours after the drug administration (20), the MCH1 mice were euthanized by cervical dislocation and their brains were collected for further molecular analysis.

### Forced-swimming test

The FST procedure was carried out according to a previously described protocol (21). Briefly, one hour following drugs or vehicle administration, mice were placed in a Plexiglass cylinder (25 cm diameter, 49 cm height) filled with water (26±1°C) to a depth of 20 cm. The mouse activity was recorded for 6 min using a camera placed in front of the cylinder and the total duration of immobility was scored as previously described (22). Immobility was considered when the mouse ceased struggling and remained floating in the water, making slight movements to keep its head above water (21). After the test, the mice were gently dried with a paper towel and returned to their cages. Latency to the first immobility and total immobility duration of the last 4 min were assessed to estimate the potential antidepressant effect of MCH1. After each test, the water was cleaned, and it was changed after four evaluations of mice from the same cage.

### Light-dark box test

The LDB test was performed to evaluate the potential anxiogenic effect of MCH1 as a possible side effect of FAAH inhibitors (23). The test was performed as previously described (24). Briefly, one hour following drugs or vehicle administration, mice were placed in a transparent (light box) Plexiglass chamber (20×20×20 cm) connected to another identical chamber with black walls (dark box). The light box was illuminated by a light source and mouse activity was recorded for 5 min. The latency of the dark box entry, the number of transitions between boxes, and the time spent in the light box were assessed to estimate the potential anxiogenic effect of MCH1. After each test, the apparatus was cleaned with 70% ethanol solution and dried.

### Open field test

The OFT was performed to assess the possible effect of MCH1 on locomotion and anxiety-like behavior (7). The protocol was followed as previously described (25). Briefly, mice were placed in a 40×40-cm arena with 50-cm-high black walls. Mouse activity and ambulation were recorded for 10 min with a camera placed 2 m above the arena. Total ambulatory distance and immobility duration were assessed using video recordings using the software Ethovision XT version 15 (Noldus, The Netherlands) to evaluate locomotor activity, while the number of entries and time spent in the center were assessed to evaluate possible anxiogenic effects. After each test, the apparatus was cleaned with 70% ethanol solution and dried.

### Tissue collection and RNA extraction

One hour after the FST, mice were euthanized and brains were extracted, cooled in dry ice, and stored at -80°C. For NAc microdissection, 500-µm coronal brain slices (Bregma + 1.42±0.2 mm) were obtained in a cryostat (CM 1520, Leica, Germany) at -10°C. Brain slices were mounted on a microscope slide over a -20°C platform inside the cryostat. Micropunches of the core zone of the NAc were then obtained with a 1.0-mm microdissector (EMS Aluminum Punch Kit, Electron Microscopy Science, USA). Immediately after, the micropunches were placed in a 1.5-mL RNAse-free tube for RNA extraction.

Total RNA was extracted from the NAc core using a modified protocol with TRIzol reagent (Invitrogen, USA) (Supplementary Material). To eliminate genomic DNA contamination, extracted RNA was incubated in DNAse (TURBO DNA-free™ kit, Invitrogen). The RNA content and purity were determined using the spectrophotometric method (NanoDrop 2000, Thermo Scientific, USA). The reverse transcription was performed on 500 ng of total RNA content using the High-Capacity cDNA Reverse Transcription Kit (Applied Biosystems, USA).

To evaluate the RNA integrity, we analyzed the *18S* normalizer gene expression in each sample using RT-qPCR (AriaMX Real-Time qPCR System, Agilent, USA). The oligonucleotides used for *18S* gene analysis were 5'-GCCGCTAGAGGTGAAATTCTT-3' (forward) and 5'-TCGTTTATGGTCGGAACTACG-3' (reverse) for the sequence accession number NR_003278.3. The protocol used was that described in BlasTaq™ 2X qPCR MasterMix (Applied Biological Materials Inc., Canada), and the expression was reported as Ct value. After that, the Ct values were compared and the samples with higher cDNA content (lower Ct) were diluted until every sample had a similar (Δ ± 1) Ct value.

### Real time quantitative PCR (RT-qPCR)

The reverse transcription product was analyzed in triplicate. Fold-change expression was assessed using the relative quantification method (ΔΔCt) (26). For that purpose, hydrolysis probes (TaqMan^®^) and AmpliTaq Gold^®^ DNA Polymerase (Thermo Fischer Scientific) were mixed with 50 ng cDNA (Supplementary Material). Information on each probe is detailed in Table 1.

**Table 1:** Information about TaqMan^®^ (Thermo Fisher Scientific) probes.

Gene	Assay ID	Amplicon length	RefSeq
*Cnr1*	Mm00432621_s1	69	MN_007726.3
*Faah*	Mm00515684_m1	62	MN_010173.4
*Drd1*	Mm01353211_m1	65	MN_010076.3
*Drd2*	Mm00438541_m1	71	MN_010077.2
*18S*	Mm03928990_g1	61	NR_003278.3

### Statistical analysis

All data, except those from OFT, were analyzed using one-way ANOVA or Kruskal-Wallis test followed by Tukey’s or Dunn's multiple comparison tests, respectively. OFT test data were analyzed using an unpaired *t*-test. All data were tested for normality using the Shapiro-Wilk test. Data with normal distribution were analyzed through a parametric test, otherwise by non-parametric tests. All data are reported as means±SE or median (IQR), and the number of subjects per group is indicated as “n”. For all tests, statistical significance was defined as P<0.05. All statistical analyses were performed using R Statistical Software (v. 4.1.2, R Foundation for Statistical Computing, Vienna, Austria) and RStudio v. 2021.09.1-372 (USA). Data plotting was performed using GraphPad Prism 9.1.2 (USA).

## Results

### Acute administration of MCH1 reduced the immobility time in the FST

**Figure 2:**
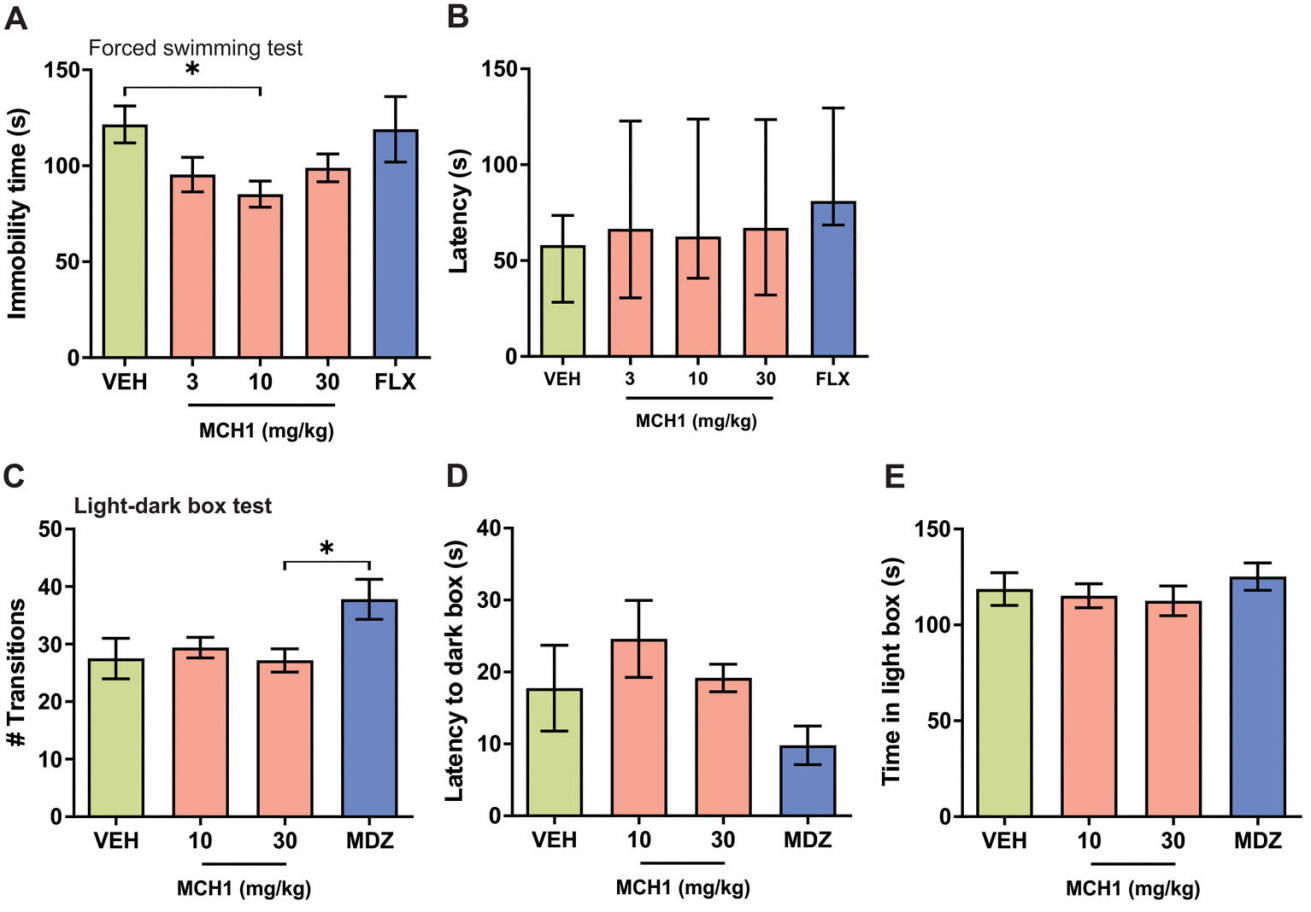
MCH1 administration showed antidepressant-like but no anxiogenic-like effect in male BALB/c mice. **A**, The group receiving 10 mg/kg MCH1 *ip* showed a reduction in the immobility time in the forced swimming test (FST) compared to vehicle. Groups receiving 3 and 30 mg/kg MCH1 and 20 mg/kg fluoxetine did not show significant differences compared to vehicle. **B**, Neither MCH1 nor fluoxetine groups showed a significant difference in the latency to first immobility in the FST compared to vehicle. Data are reported as median (±IQR). **C**, None of the MCH1 doses showed a significant difference compared to vehicle in the number of transitions between boxes in the light-dark box (LDB). However, there was a significant difference between the positive control 2 mg/kg midazolam and 30 mg/kg MCH1. None of the MCH1 doses nor the midazolam group showed a significant difference compared to vehicle in the latency to dark box (**D**) or time spent in the light-box (**E**) in the LDB test. Data are reported as means±SE. *P<0.05, Tukey's HSD test. VEH: vehicle; FLX: fluoxetine; MDZ: midazolam; MCH1: (9Z,12Z)-N-benzyloctadeca-9,12-dienamide.

The three groups that received MCH1 showed a tendency to reduce the immobility time compared to the VEH group (Figure 2A). One-way ANOVA analysis showed a significant difference among the groups [F_(4,53)_=2.84, P=0.0332]. Tukey's multiple comparison *post hoc* analysis revealed that only the 10 mg/kg MCH1 group was significantly different compared to the VEH group (P<0.05). Although the 3 and 30 mg/kg MCH1 groups also showed a tendency to reduce the immobility time compared to the vehicle group, this reduction was not significant (P>0.05). The fluoxetine group also did not show a significant difference compared to the VEH (P>0.05). Latency analysis for the first episode of immobility showed no significant difference among the groups (K=5, P=0.4027, Kruskal-Wallis test) (Figure 2B).

### Acute administration of MCH1 did not alter anxiety-like behavior in the LDB test

None of the parameters evaluated in the LDB test showed an effect on MCH1. Analysis of the number of transitions between chambers (F_(3,16)_=3.462, P=0.0413; one-way ANOVA) showed a significant increase only in the MDZ group compared to MCH1 30 mg/kg (P<0.05, Tukey's multiple comparison test) (Figure 2C). The latency to entry to the dark box (F_(3,16)_=2.40, P>0.05; one-way ANOVA) (Figure 2D) and the time spent in the light compartment (F_(3,16)_=1.78, P>0.05; one-way ANOVA) (Figure 2E) were not significantly different. These results suggest no anxiogenic effects of MCH1.

### Acute administration of MCH1 increased locomotion with no anxiogenic effect in the OFT

**Figure 3:**
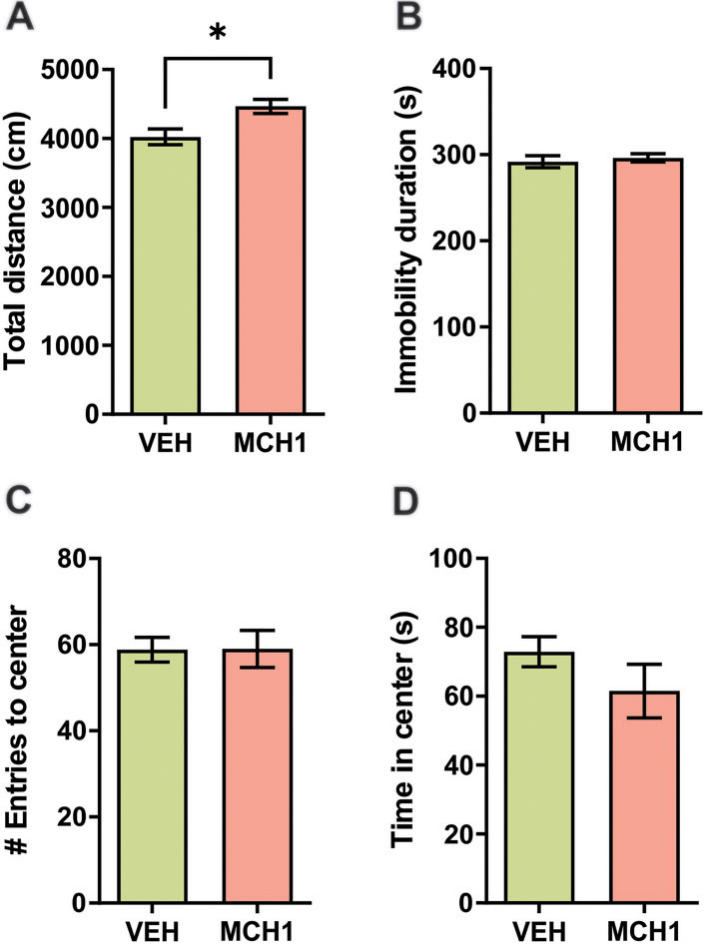
MCH1 increases locomotion but not anxiogenic-like behavior in male BALB/c mice. **A**, MCH1 10 mg/kg increased the total ambulatory distance significantly compared to vehicle in the open field test (OFT), but the MCH1 did not have a significant effect on the total immobility time during the 10-min evaluation (**B**). MCH1 also did not show an anxiogenic-like effect assessed by the number of entries into the center (**C**) and the time spent in the center of the arena (**D**) in the OFT. Data are reported as means±SE. *P<0.05, *t*-test. MCH1: (9Z,12Z)-N-benzyloctadeca-9,12-dienamide.

The administration of 10 mg/kg MCH1 significantly increased total ambulatory distance of mice (*t*_(17)_=2.89, P=0.010; unpaired *t*-test) (Figure 3A) with no change in immobility duration (*t*_(17)_=0.530, P=0.604; unpaired *t*-test) (Figure 3B), number of entries (*t*_(17)_=0.039, P=0.970; unpaired *t*-test) (Figure 3C), or time spent in the center (*t*_(17)_=1.27, P=0.226; unpaired *t*-test) (Figure 3D).

### Acute administration of MCH1 increased the mRNA expression of *Faah* and *Cnr1* in the NAc

**Figure 4:**
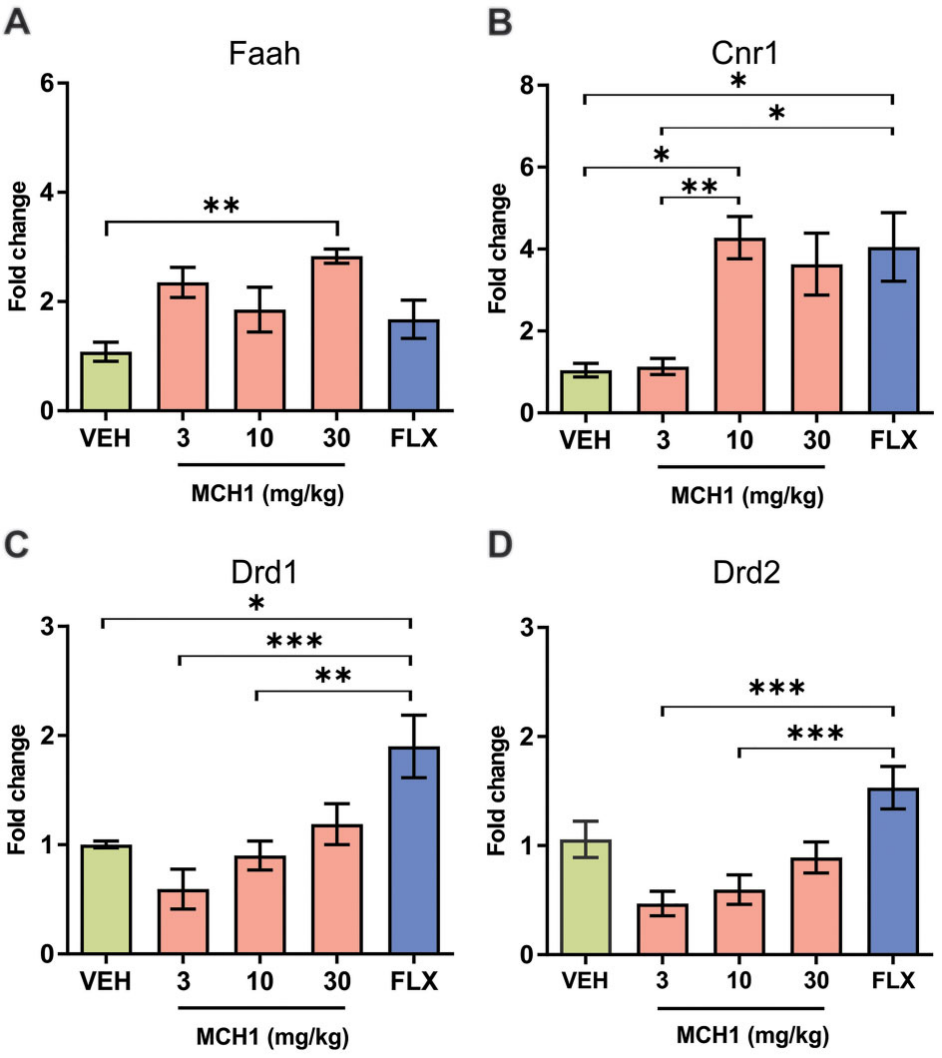
MCH1 administration increases *Faah* and *Cnr1*, but not *Drd1* or *Drd2* mRNA expression in nucleus accumbens of male BALB/c mice. **A**, MCH1 30mg/kg led to an increase of *Faah* mRNA expression up to 2.8-fold compared to vehicle. Although 3 and 10 mg/kg tended to increase *Faah* mRNA expression, this increase was not significantly different from vehicle. **B**, MCH1 10 mg and fluoxetine (FLX) groups showed an increase in *Cnr1* mRNA expression up to 4.3-fold compared to vehicle. Significant differences were also observed between 3 and 10 mg/kg MCH1 groups and 3 mg/kg MCH1 and FLX group. **C**, No significant difference between any MCH1 group and vehicle in *Drd1* mRNA expression was observed. However, the fluoxetine group showed an increased *Drd1* mRNA expression compared to vehicle (1.9-fold change), MCH1 3 mg/kg, and MCH1 10 mg/kg. **D**, In the *Drd2* mRNA expression analysis, no significant differences were observed between any MCH1 dose and vehicle. Only the fluoxetine group showed a significant increase in *Drd2* mRNA expression compared to MCH1 3 and 10 mg/kg. Data are reported as means±SE. *P<0.05, **P<0.01, and ***P<0.001, Tukey's HSD test. MCH1: (9Z,12Z)-N-benzyloctadeca-9,12-dienamide.

MCH1 has been described as an inhibitor of the FAAH enzyme. We measured the expression levels of the *Faah* gene in the NAc of animals acutely treated with MCH1. Two hours after MCH1 administration, *Faah* expression changed significantly in the NAc (F_(4,31)_=3.94, P=0.011; one-way ANOVA). Tukey's multiple comparison *post hoc* analysis revealed that 30 mg/kg MCH1 increased the expression of the VEH group significantly by 2.8-fold (P=0.009). Although the 3 and 10 mg/kg MCH1 doses showed an increasing trend in *Faah* expression compared to the VEH group (2.4- and 1.9-fold, respectively), this increase was not statistically significant (P>0.05). No change in *Faah* expression was found in the FLX group (P>0.05) (Figure 4A).

To examine whether the acute administration of MCH1 could have some influence on cannabinoid signaling function in the NAc, we measured the gene expression of the CB1 receptor. One-way ANOVA showed that acute administration of MCH1 significantly changed the expression of the *Cnr1* gene in the NAc (F_(4,29)_=5.56, P=0.002) (Figure 4B). Indeed, Tukey's multiple comparison test revealed that 10 mg/kg MCH1 increased the *Cnr1* expression by 4.3-fold compared to VEH (P<0.05). No significant change in gene expression was found at the 3 and 30 mg/kg MCH1 doses (P>0.05). Interestingly, the FLX group also showed a 4.1-fold gene expression increase compared to the VEH group (P<0.05).

### Acute administration of MCH1 did not change the *Drd1* and *Drd2* mRNA expression in NAc

To investigate whether the acute administration of MCH1 could have some influence on dopaminergic signaling function in the NAc, we measured the gene expression of the D1 and D2 receptors. One-way ANOVA showed a significant change in the *Drd1* mRNA expression among the groups (F_(4,33)_=6.99, P=0.0003). However, *post hoc* Tukey's multiple comparison test revealed that none of the MCH1 doses differed significantly from *Drd1* mRNA expression levels measured in the VEH group (P>0.05). Indeed, 3, 10, or 30 mg/kg of MCH1 increased expression levels by 0.6-, 0.9-, and 1.2-fold, respectively, in the VEH group. Interestingly, the VEH, 3, and 10 mg/kg MCH1 groups showed significantly reduced *Drd1* mRNA expression levels compared to the FLX group (P<0.05) (Figure 4C).

When comparing the *Drd2* mRNA expression in the NAc, a significant difference was found (F_(4 33)_=8.03, P=0.0001; ANOVA). However, *post hoc* Tukey's multiple comparison test showed that neither MCH1 dose induced a significant change in the *Drd2* mRNA expression compared with the VEH group. The 3, 10, and 30 mg/kg doses increased gene expression levels by 0.5, 0.6, and 0.9-fold of the VEH group (P>0.05). The expression level of the FLX group did not differ from the VEH group, however, expression levels of the 3 and 10 mg/kg MCH1 groups were significantly lower than those of the FLX group (P<0.05) (Figure 4D).

## Discussion

In this study, we demonstrated that acute administration of 10 mg/kg MCH1 significantly reduced immobility time in the FST and the 30 mg/kg dose increased the expression of endocannabinoid signaling-related genes (*Cnr1* and *Faah*), with no changes in dopamine signaling genes (*Drd1* and *Drd2*) in the NAc. We hypothesized that this new FAAH inhibitor, tested in *in-vitro* studies (2,27), could potentially have an antidepressant-like effect when acutely administrated in a living mouse. While the 10 mg/kg dose decreased the immobility time, the 3 and 30 mg/kg doses (minimum and maximum, respectively) caused a non-significant reduction. This phenomenon is known as a U-shaped dose-response curve, which is typical of endocannabinoid drugs (28). In fact, CB1 agonists like cannabidiol (29) and FAAH inhibitors like AA-5HT (30) have similar effects. Kirkedal et al. (30) hypothesized that this phenomenon could be attributed to the capacity of anandamide to act on receptors other than CB1. A low dose of FAAH inhibitor may not be enough to elicit a significant behavioral response, as anandamide levels increase only slightly. However, a high dose of FAAH inhibitor could increase anandamide levels leading to activation of the TRPV1 channel. TRPV1 channel activation is associated with depressive-like behavior induction and could attenuate the potential antidepressant-like effect of the endocannabinoid drug.

In addition, 10 mg/kg MCH1 did not induce an anxiolytic-like effect as measured in the LDB. Our results of the OFT also revealed that the mice traveled a greater distance in the arena without a significant effect in the anxious-like behavior. In studies of behavioral tests for screening new antidepressant drugs, reduced FST immobility time is interpreted as reduced depressive-like behavior, as the mouse remains less time in a state of “behavioral despair' in the face of inescapable stress (31). In this study, we used an acute dose of the selective serotonin reuptake inhibitor (SSRI) fluoxetine (20 mg/kg, *ip*) as a positive control, as it reduces the behavioral despair in the FST 30-60 min after a single injection (32). Interestingly, we saw no significant behavioral changes in the fluoxetine group compared to the vehicle group. Indeed, in another independent study in the same inbred mice, we did not find any effect of a single injection of 10, 20, or 40 mg/kg (*ip*) of fluoxetine on the immobility induced by FST (data not shown). Since the positive control showed no effect on immobility induced by FST, it is therefore difficult to interpret our results of reduction in immobility time by the 10 mg/kg dose of MCH1 as a potential antidepressant effect. However, new understandings have considered that FST findings could be evaluated as a coping strategy for inescapable stressors rather than depression-like behavior (33,34). From this approach, reduction in immobility time and increase in locomotor activity induced by acute administration of 10 mg/kg MCH1 could be interpreted also as an enhanced coping strategy in the FST.

FAAH hydrolyzes endocannabinoids, especially AEA (3), and inhibiting its activity leads to an increased concentration of AEA in the central nervous system even with a single dose (35) and in *in vitro* assays. Given that MCH1 has been shown to inhibit the FAAH enzyme activity irreversibly, we hypothesized that administration of MCH1 in mice could induce an antidepressant-like effect through changes in the endocannabinoid signaling in the NAc, specifically in the CB1 receptor and FAAH enzyme genes. Indeed, we found that 10 and 30 mg/kg MCH1 doses increased *Cnr1* mRNA expression in the NAc core. This acute response can be explained by the hypothetical increase of AEA bioavailability following irreversible FAAH blockage and CB1 receptor activation. *In vitro* evidence suggests that CB1 activation can increase *Cnr1* mRNA expression via extracellular signal-regulated kinases 1 and 2 (ERK1/2) signaling (36). Our results are are in agreement with previous studies showing an increase of *Cnr1* mRNA expression after 1.5 h of URB597 administration in a pain perception model in rats, although this was evidenced in the mesencephalon, thalamus, and hypothalamus but not in the striatum (37). Another study also revealed an increase in *Cnr1* mRNA expression in the hippocampus after 2 weeks of URB597 treatment in a chronic constriction injury pain model in rats (38). Interestingly, Rivera et al. (20) observed that after a single URB597 administration followed by a 4-day resting period, *Cnr1* mRNA expression in striatum was reduced. These contradictory results can be attributed to the model used, the reversibility of the FAAH blockage, and the time of tissue collection. Future studies must be conducted to explore the dynamics of *Cnr1* mRNA expression after an acute FAAH inhibitor administration.

As MCH1 is an irreversible inhibitor of the FAAH enzyme activity, we expected a reduced effect on the *Faah* genes. However, we found that two hours after *ip* injection and one hour after FST, a 30 mg/kg MCH1 dose increased the expression of the *Faah* gene. A possible explanation for this finding is that the irreversible inhibition of FAAH enzyme by MCH1 could lead to an internalization of the intracellular membrane-bound enzyme and FAAH depletion. Thus, in order to maintain homeostasis, striatum neurons might increase *Faah* mRNA expression to compensate for this depletion. However, a previous study has shown that an acute (brain collected after four days of a single-dose administration) or repeated administration of URB597, a reversible FAAH inhibitor, increased *Faah* mRNA expression, but not the protein expression in the striatum (20), suggesting that *Faah* gene expression changes are relatively stable over time. Future studies must be performed to assess FAAH protein expression after acute MCH1 administration and evaluate the transient changes in *Faah* gene expression.

It was observed that the administration of MCH1 did not significantly modify the expression of the *Drd1* and *Drd2* genes in the vehicle group. However, a tendency to decrease their expression was observed with the 3 mg/kg dose and their expression increased thereafter with increasing doses until the 30 mg/kg dose reached levels similar to those of the vehicle. To date, there is no published evidence about the effect of acute administration of FAAH inhibitors on *Drd1* or *Drd2* mRNA expression in the NAc. On the other hand, a significant increase in the expression of *Drd1* and *Drd2* was found in the fluoxetine group compared to the lower dose MCH1 groups or the vehicle. No information is currently available regarding the effect of fluoxetine in an acute stress model. Furthermore, chronic administration of fluoxetine and sertraline, another selective serotonin reuptake inhibitor, has been reported to increase the expression of the *Drd1* gene (39) in the NAc and hippocampus (40), although the mechanism of such increase has not yet been described.

In summary, our study concluded that the irreversible FAAH inhibitor MCH1 affects the endocannabinoid system, but not dopaminergic signaling-related genes in the NAc. MCH1 also shows a potential antidepressant-like effect, but no anxiogenic side effects in *in vivo* models. This last conclusion must be taken carefully due to the influence of the increased locomotor activity.

A limitation of the study is the assumption that the mechanism of action of the macamide is through the inhibition of FAAH and an increase in anandamide bioavailability, as shown in previous *in vitro* studies. However, in a complex system such as a living animal, MCH1 could be acting not only through the endocannabinoid pathway but also through unexpected pathways. One way to test this hypothesis would be through the use of endocannabinoid receptor antagonists, which would act as negative controls for the therapeutic effect of macamides. Another important limitation concerns the level of inference that can be drawn from molecular experiments. The changes in gene expression allow us to observe the early changes resulting from experimental interventions. However, they do not necessarily correlate directly or linearly with the neurobiological changes responsible for the behavioral alterations. Neurobiological changes such as morphological and electrophysiological changes follow genetic changes, so it is necessary to verify the existence of this theoretical relationship between both aspects.

## Supplementary Material

Click to view [pdf].
