# Supplementary material for: Effect of MCH1, a fatty-acid amide hydrolase inhibitor, on the depressive-like behavior and gene expression of endocannabinoid and dopaminergic-signaling system in the mouse nucleus accumbens
Source: Braz J Med Biol Res. 2024 Feb 19;57:e12857. doi: 10.1590/1414-431X2024e12857 (PMC10880885; doi:10.1590/1414-431X2024e12857)
Supplement: Supplementary file 1 [file 1414-431X-bjmbr-57-e12857-suppl.pdf]

SUPPLEMENTARY MATERIAL

TOTAL RNA EXTRACTION USING A MODIFIED PROTOCOL WITH TRIZOL REAGENT

The TRIzol™ methodology with modifications was used for RNA extraction from the microdissected samples. Tissue samples (1 mg approximately) were immediately transferred to 1.5 mL nuclease-free cone tube on ice. Then, 200 µL of Mammalian Lysis Buffer and 2 µL of ribonuclease inhibitor (RiboLock RNase inhibitor, Thermo Fischer) were added and the mixture was homogenized using a 1mL syringe. After that, 300 µL of TRIzol™ reagent (Invitrogen) was added and the tube was vigorously shaken for 15 seconds and allowed to stand at room temperature for 5 minutes. After the incubation, 200 µL of chloroform was added, shaken for 15 seconds, and allowed to stand for 3 more minutes. The mixture was then centrifuged at 13700 *g*, 4°C for 15 minutes and with the help of a 20-200 µL micropipette, the supernatant was transferred to another 1.5 mL nuclease-free tube, which was placed in a box with ice. In the second tube, 200 µL of chloroform was added, shaken for 15 seconds, and centrifuged at 13700 *g*, 4°C for 5 minutes. Then, using a 20-200 µL micropipette, the supernatant was transferred to a final 1.5 mL tube free of nucleases. 500 µL of molecular grade isopropanol and 3.5 µL of co-precipitant (GlycoBlue-TM Blue Co-precipitant, Invitrogen) were added to the tube and allowed to stand at -20°C for 30 minutes. The tube was then removed from the refrigerator and centrifuged at 13700 *g*, 4°C for 15 minutes. A blue precipitate was observed at the bottom of the tube and the isopropanol was carefully removed by tilting the tube, preventing the precipitate from falling off. After that, 1000 µL of 75% molecular-grade ethanol was added to the tube and then vortexed for 15 seconds and centrifuged at 5400 *g*, 4°C for 5 minutes. Again, the solvent was carefully removed to avoid dislodging the precipitate and 1000 µL of 75% molecular grade ethanol was added again. The tube was vortexed for 15 seconds and then centrifuged at 5400 *g*, 4°C for 5 minutes. For the final step, the solvent was removed and centrifuged using a minicentrifuge to settle the last drops of ethanol. These drops were removed by extracting them with a 1-10 µL micropipette. Then, 11 µL of DEPC water was added. The tube was shaken for 15 seconds in the vortex to dissolve the precipitate and incubated at 65°C for 5 minutes to remove secondary RNA structures before quantification. It was shaken again in the vortex and centrifuged in the spinner. Finally, the tube was placed on ice until quantification. The quantification was performed using the spectrophotometric method (NanoDrop 2000, Thermo Scientific). First, the instrument was set to RNA reading mode and 1 µL DEPC water was used to record the baseline. Then 1 µL of RNA sample was placed on the pedestal and measured absorbance in the range of 200 to 600 nm. The UV spectrum, the estimated amount in ng/µL and the 280/260 and 230/260 ratios that are indicative of the presence of protein and mineral impurities, respectively, were recorded. An acceptable sample was defined as any sample showing concentrations greater than 100 ng/µL and with a 280/260 ratio greater than 1.8. Before performing retro-transcription, it was necessary to confirm that all genomic DNA was removed. For this purpose, the endpoint PCR technique was employed using the Amplitronyx ™ 6 thermocycler (NYX Technik) and the DreamTaq DNA polymerase reagent kit (Thermo Scientific). The cleaning procedure and removal of genomic DNA contamination were performed by DNase treatment (TURBO DNA-free™ kit, Invitrogen).

RNA extraction workflow

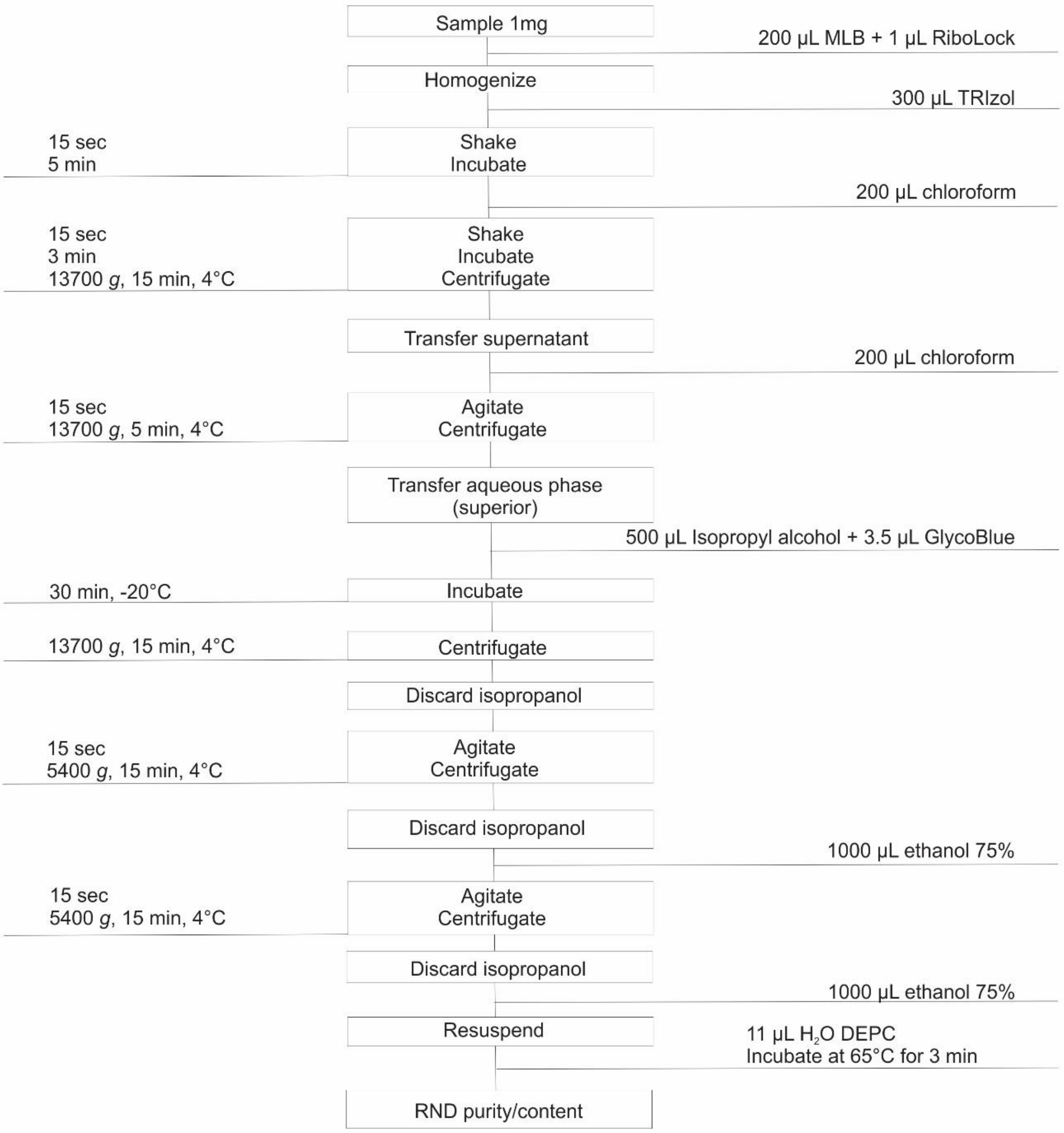

REAL-TIME QUANTITATIVE PCR WITH HYDROLYSIS PROBES (TaqMan®)

RT-qPCR technique with hydrolysis probes was used for the gene expression analysis of *Faah*, *Cnr1*, *Drd1* and *Drd2*. For that purpose, we used the commercial kit AmpliTaq Gold™ DNA Polymerase with Buffer II and $MgCl_2$ (Thermo Fischer). Relative quantification was selected as a method for comparing experimental groups. First, the 2X Master Mix with the DNA polymerase for amplification was prepared as described in Table S1.

Table S1. Master Mix 2X components for qPCR with hydrolysis probes

Reactant	Concentration	Volume (µL)
Buffer	10 X	200
$MgCl_2$	25 µM	120
dNTPs	10 mM	40
ROX	50 X	40
AmpliTaq Gold™ polymerase	5 U/µL	10
Free-RNAse H_2O	-	590
Total volume		1000

Next, the reaction mix was prepared as described in Table S2. Each TaqMan® 20X Probe contained the FAM-MGB dye and the Forward/Reverse primers of the gene to be analyzed. Each sample was analyzed in duplicate.

Table S2. Components of the PCR amplification reaction with TaqMan® probes

Reactant	Concentration	1X - Volume (µL)
Master Mix 2X	2 X	5
TaqMan® probes	20 X	1
Free-RNAse H_2O	10 mM	2
cDNA	25 ng/µL	2
Total volume		10

Duplicate samples were placed in the real-time thermal cycler and analyzed using the FAM optical module. The thermal cycler temperature program is detailed in Table S3.

Table S3. Real-time thermocycler program for PCR amplification with TaqMan® probes

Phase	Temperature (°C)	Duration (min)
Hot Start	50	2:00
	95	10:00
Amplification (40 cycles)	95	0:15
	60	1:00

The qPCR technique with hydrolysis probes does not produce a melting curve, so that step was excluded from the temperature program. For each sample, the 6 genes were analyzed in different tubes, in addition to the normalizing gene (18S).
